# Catching Cold

**Published:** 1897-02

**Authors:** 


### CATCHING COLD.

The "cold spots," meaning thereby the surface areas peculiarly susceptible to cold, are principally the nape of the neck and the lower part of the back of the head, the front of the abdomen and the shins. The acute discomfort and the sense of impending disaster which result from the steady play of a current of cold air upon the neck from behind are well known. The necessity of keeping the abdomen warmly clad is also generally recognized, though perhaps not as generally carried into practice. Curiously enough, few people are conscious of the danger they run by exposing the usually inadequately protected shins to currents of cold air. This is the usual way in which colds are caught on omnibuses. When driving one takes care to cover the legs with a rug or waterproof, but on the more democratic conveyance rugs are not often available and the reckless passenger by and by awakens to the fact that the iron has entered his soul—in other words, that he has "caught cold." People who wear stockings, such as Highlanders, golfers and cyclists, invariably take the precaution of turning the thick woolen material down over the shins, the better to protect them against loss of heat, though, incidentally the artificial embellishment of the calves may be altogether foreign to the maneuvre. This is an instance of how all things work together for good. It does not, of course, follow, because certain areas are peculiarly susceptible to cold, that a chill may not be conveyed to the nervous system from other points. Prolonged sitting on a stone, or even on the damp grass, is well known to be a fertile source of disease; and wet cold feet are also, with reason, credited with paving the way to an early grave.—*London Medical Press.*

